# Statistical Study of the Properties of Magnetosheath Lion Roars

**DOI:** 10.1029/2018JA025343

**Published:** 2018-07

**Authors:** Stefanos Giagkiozis, Lynn B. Wilson, James L. Burch, Olivier Le Contel, Robert E. Ergun, Daniel J. Gershman, Per-Arne Lindqvist, Laurent Mirioni, Thomas E. Moore, Robert J. Strangeway

**Affiliations:** 1 Automatic Control and Systems Engineering, University of Sheffield, Sheffield, UK,; 2 Heliophysics Science Division, NASA Goddard Spaceflight Center, Greenbelt, MD, USA,; 3 Southwest Research Institute, San Antonio, TX, USA,; 4 Laboratoire de Physique des Plasmas, UMR7648, CNRS, Ecole Polytechnique, Sorbonne Universités, Université Paris-Sud, Observatoire de Paris, Paris, France,; 5 Laboratory for Atmospheric and Space Physics, University of Colorado Boulder, Boulder, CO, USA,; 6 Fields and Particles, NASA Goddard Space Flight Center, Greenbelt, MD, USA,; 7 Department of Astronomy, University of Maryland, College Park, MD, USA,; 8 Department of Space and Plasma Physics, KTH Royal Institute of Technology, Stockholm, Sweden,; 9 Laboratoire de Physique des Plasmas (LPP), CNRS/ Ecole Polytechnique/ Sorbonne Universités UPMC/ Paris-Sud 11, Palaiseau, France,; 10 Institute of Geophysics and Planetary Physics and Earth and Space Sciences, University of California, Los Angeles, CA, USA

## Abstract

Lion roars are narrowband whistler wave emissions that have been observed in several environments, such as planetary magnetosheaths, the Earth’s magnetosphere, the solar wind, downstream of interplanetary shocks, and the cusp region. We present measurements of more than 30,000 such emissions observed by the Magnetospheric Multiscale spacecraft with high-cadence (8,192 samples/s) search coil magnetometer data. A semiautomatic algorithm was used to identify the emissions, and an adaptive interval algorithm in conjunction with minimum variance analysis was used to determine their wave vector. The properties of the waves are determined in both the spacecraft and plasma rest frame. The mean wave normal angle, with respect to the background magnetic field (**B**_0_), plasma bulk flow velocity (*V*_*b*_), and the coplanarity plane (**V**_*b*_ × **B**_0_) are 23°, 56°, and 0°, respectively. The average peak frequencies were ~31% of the electron gyrofrequency (*ω*_*ce*_) observed in the spacecraft frame and ~18% of *ω*_*ce*_ in the plasma rest frame. In the spacecraft frame, ~99% of the emissions had a frequency < *ω*_*ce*_, while 98% had a peak frequency < 0.72 *ω*_*ce*_ in the plasma rest frame. None of the waves had frequencies lower than the lower hybrid frequency, *ω*. From the probability density function of the electron plasma *β*_*e*_, the ratio between the electron thermal and magnetic pressure, ~99.6% of the waves were observed with *β*_*e*_ < 4 with a large narrow peak at 0.07 and two smaller, but wider, peaks at 1.26 and 2.28, while the average value was ~1.25.

## 1. Introduction

The Earth’s magnetosheath lies between the bow shock and the magnetopause where the solar wind is decelerated to subsonic speeds and is diverted around the magnetopause. The plasma properties of the region depend strongly on the solar wind conditions, the magnetic field upstream of the extended bow shock, and the angle of the shock normal vector with respect to the upstream average magnetic field (*θ*_*Bn*_). The magnetic field in the sheath is strongly perturbed by several sources of turbulence and instabilities (see Lucek et al., 2005, for a review).

Packets of short duration (few seconds), right-hand polarized waves, and frequency less than a few hundred hertz are very common in the magnetosheath (Smith et al., 1969). Smith et al. (1969) called this type of emission *lion roars* (LRs)—identified as whistler mode waves—because of their sonified resemblance to male African lion calls. In the majority of studies, the propagation angle relative to the background magnetic field is *θ*_*kB*_ < 30° (Baumjohann et al., 1999; Smith &Tsurutani, 1976;Tsurutani et al., 1982), while Zhang et al. (1998) observed LRs with a much wider range of wave normal angles, some of them, mainly in the vicinity of the bow shock, that were highly oblique. Zhang et al. (1998) also used the evolution of the propagation direction within an interval with LR packets to determine the distance of the spacecraft from the source of the emissions.

Smith and Tsurutani (1976) found a correlation between LR observations and decreases in the magnetic field intensity, accompanied by an increase in particle density. These magnetic field decreases have been linked to mirror mode structures (Vedenov & Sagdeev, 1961)—the result of an instability where the thermal pressure is anticorrelated with magnetic field fluctuations. Baumjohann et al. (1999) performed a statistical study of LRs associated with mirror modes, using 128 Hz magnetic field measurements from Equator S. The observations showed a typical frequency range of [0.05–0.15]*f*_*ce*_. They argued that due to the confinement of the electrons in a mirror mode, a limit will be imposed on their perpendicular velocity and therefore the region of resonance in the electron velocity space would be limited, compared to the narrow strip shown by Kennel and Petschek (1966) for other cases. The relatively large wave intensities of whistlers would then lead to electron diffusion toward the parallel velocity direction (Baumjohann et al., 1999). This would result in a distortion in the contours at the region of resonance similar to the one shown by Kivelson and Southwood (1996). A case study of LR emissions in mirror mode structures has been performed by Breuillard et al. (2017), where the properties and dynamics of the waves have been examined. The authors also argue that a statistical study with higher-frequency magnetometer data could potentially reveal higher amplitude in the amplitudes of the measurements, compared to the ones provided by Baumjohann et al. (1999).

Tsurutani et al. (1982) concluded that LRs observed close to the magnetopause are generated by the electron cyclotron instability when *T*_⊥*e*_ > *T*_∥*e*_ and a decrease in the magnetic field. The decrease in the magnetic field and the increase of the density, related to the mirror mode, cause the local plasma critical energy (*E*_crit_ = *B*^2^*/*8*πN*), where *N* is the density and *B* the magnetic field, to drop close to the value of the electron thermal energy. When the magnetic field increases, this is no longer true and so the emission terminates. LRs though are not always accompanied by a dip in the magnetic field (e.g., see Zhang et al., 1998). Kennel and Petschek (1966) showed that the stability of whistler mode waves depends on the electron temperature anisotropy *A*_*e*_ = *T*_⊥*e*_*/T*_∥*e*_ − 1, under the condition $\frac{\omega }{\Omega_{ce}}<\frac{A_{e}}{A_{e}+1}$. The growth or damping of the waves depends also on the resonant frequency of the electrons when compared to the plasma critical energy (*E*_*r*_ > *E*_crit_). Masood et al. (2006) used this result to show that the majority of the observed magnetosheath LRs in the study originated from a remote region, since there was no correlation with *A*_*e*_, as LRs existed in all cases where *A*_*e*_ < 0, *A*_*e*_ ~ 0, and *A*_*e*_ > 0. Wilson et al. (2013) showed that when the entire distribution is used, less than half of the observed whistler waves satisfied the instability inequality, while 75% satisfied the inequality when only the halo was considered. Wilson et al. (2013) show an example electron distribution where they compute *A*_*e*_ for the entire, core, and halo components separately. They found *A*_*e*_ = −0.04, −0.08, and +0.25 for the entire, core, and halo components, respectively. The core and entire components do not satisfy the inequality but the halo does. The reason is that the typical cyclotron energies correspond to the halo, not the core (Wilson et al., 2013).

Using STEREO measurements, Breneman et al. (2010) observed narrowband whistler waves, mainly within stream interaction regions and to a smaller extent, in the vicinity of interplanetary shocks. The authors determined that the emissions had similar characteristics to LRs observed in the magnetosheath. Using the wave parameters observed, Breneman et al. (2010) performed particle tracing simulations which showed a strong interactions of whistler mode waves with halo electrons. The simulations show the largest pitch angle and energy diffusion for a wave normal angle of 45°. The particles used in the simulation were at 100 eV and a 75° pitch angle, and the whistlers had a 10 mVm^−1^ amplitude and variety of propagation angles.

In section 2 the measurements, data sets, and methodology used for the study will be presented. An example LR that was identified by the semiautomatic algorithm is also presented. In section 3 the statistical results of the study are presented. In section 4 a discussion on the properties and statistical results is presented and a final conclusion is given.

## 2. Data and Methodology

Magnetic field (**B**) measurements from the Magnetospheric Multiscale (MMS) search-coil magnetometer (SCM) (Le Contel et al., 2016) were mainly used to identify and study the properties of LRs. SCM measurements were used only during burst mode operation of the spacecraft when **B** is sampled at 8,192 Hz. The sensitivity of the SCM is $\frac{2\text{ pT}}{\sqrt{\text{HZ}}}$ at 10 Hz, $\frac{0.3\text{ pT}}{\sqrt{\text{Hz}}}$ at 100 Hz, and $\frac{0.05\text{ pT}}{\sqrt{\text{Hz}}}$ at 1 kHz. At 1 kHz the resolution is 0.15 pT. The SCM data that were used in the study were high-pass filtered at a 10-Hz cutoff frequency. Measurements with amplitude smaller than 5-pT peak to peak were also not considered. The quasi-static magnetic field (**B**_0_) was obtained by the fluxgate magnetometer (Russell et al., 2016), which provides measurements up to 128 vectors/s. Particle number density (*n*) and bulk velocity (**V**_*b*_) were obtained by the fast plasma investigation (Pollock et al., 2016), which can provide measurements of 3-D ion and electron distributions at 150- and 30-ms time resolution, respectively. The electric field, **E**, is measured by the spin-plane double probe and the axial double probe instruments on board the MMS (Torbert et al., 2016) and provides the same sampling as **B**_0_. The parameters of the magnetopause (Farris et al., 1991) and bow shock (Farris & Russell, 1994) models used in the paper were obtained from Schwartz (1998).

The **B** measurements were rotated to the field-aligned coordinate (FAC) system, not to be confused with field-aligned currents usually denoted as FAC as well. The first coordinate of the FAC system of reference (${\hat{e}}_{\|}$) points in the direction of **B**_0_. The second coordinate is defined as ${\hat{e}}_{⊥2}={\hat{e}}_{\|}\times x$, where **x** = [1, 0,0] (the *x* direction of the GSE coordinate system). The third coordinate completes the orthogonal basis and is defined as ${\hat{e}}_{⊥1}={\hat{e}}_{⊥2}\times {\hat{e}}_{\|}$.

The presence of transverse waves was automatically determined, by looking where the magnitude of **B**_0_ in the ${\hat{e}}_{\|}$ direction is smaller than the magnitude of **B**_0_ in the ${\hat{e}}_{⊥1}$ and ${\hat{e}}_{⊥2}$ directions. LRs could then be found by looking for a relatively narrowband peak in the power spectra of the intervals containing transverse waves.

**Figure 1.**
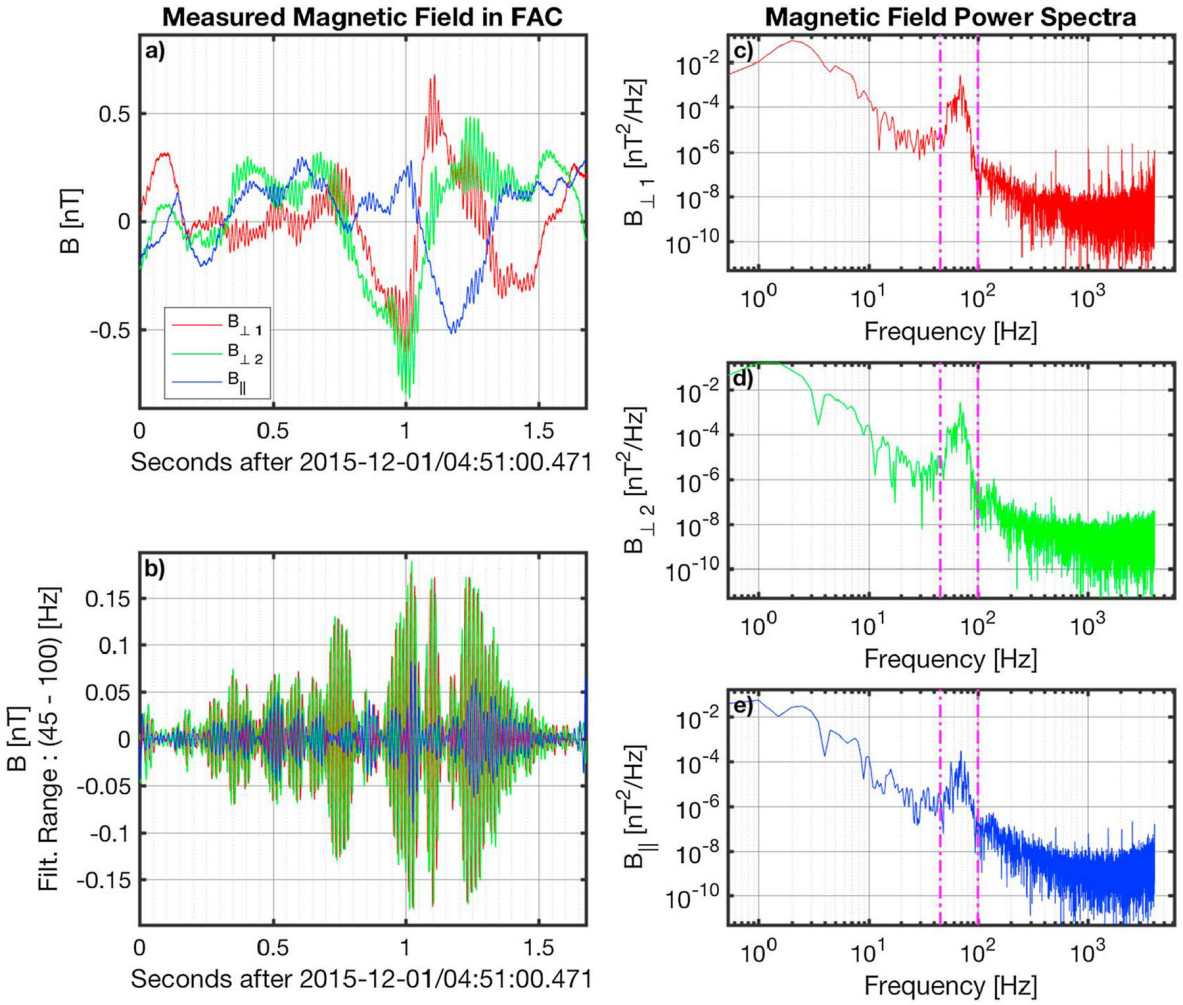
Magnetic field measurements of an example Lion roar. The magnetic field in FAC is shown in panel a. The band-pass ([45–100] Hz) filtered signal of the emission is shown in panel b. Panels c-e show the power spectrum for each of the three magnetic field components **B**_⊥1_, **B**_⊥2_, and **B**_∥_, respectively. The vertical magenta lines in panels c-e denote the frequency band of the emission as identified by the power spectra. FAC = field-aligned coordinates.

**Figure 2.**
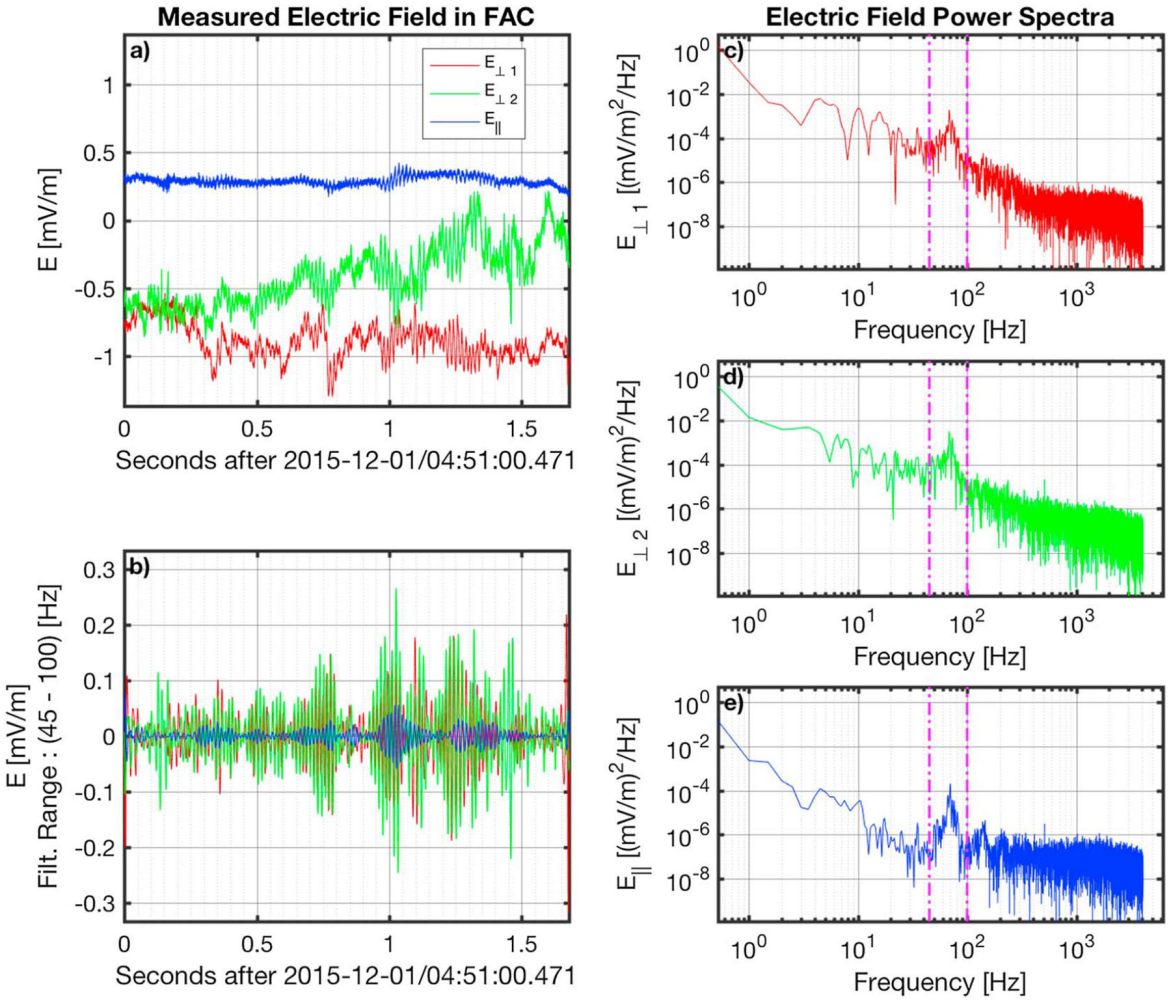
Electric field measurements of an example lion roar. The measured electric field in FAC is shown in panel a. The band-pass ([45–100] Hz) filtered signal of the emission is shown in panel b. Panels c-e show the power spectrum for each of the three electric field components **E**_⊥1_, **E**_⊥2_, and **E**_∥_, respectively. The vertical magenta lines in panels c-e denote the frequency band of the emission as identified by the power spectra. FAC = field-aligned coordinates.

An example of an observed LR is shown in Figure 1. Panel a shows the measured **B** in FAC, while panel b shows the band-pass filtered signal of the same interval. The LR was observed by MMS3 on 1 December 2015 at 4:51:00.471 UT, and the emission lasted for about 1.6 s. The bandwidth of the filter was determined from the peak in the power spectra (Figures 1c–1e) of each of the three components. In this example the frequency band of the emission was identified to be between 45 and 100 Hz and the average peak frequency between the three FAC directions is 69 Hz. The electric field measurements of the interval are also shown in Figure 2, which has the same format with Figure 1. The peak in the electric field is also within the same bandwidth as in the case of the magnetic field. The combined filtered time-series of the magnetic and electric fields show that the wave has an electromagnetic nature.

The individual LR intervals were then analyzed using a minimum variance analysis (MVA) with adaptive interval selection (e.g., see Wilson III et al., 2017, for discussion). The software splits the original interval into smaller subintervals which maximize the intermediate to minimum (*λ*_int_/*λ*_min_) eigenvalue ratio and minimize the maximum to intermediate (*λ*_max_/*λ*_int_) ratio. In order to achieve that, the original interval is split into smaller overlapping subintervals of variable length and MVA is applied to each of those subintervals. The intervals that are kept are the ones that best satisfy the objectives. In order to ensure that the waves that were observed were whistlers and to ensure that they were circularly polarized, only subintervals for which *λ*_int_/*λ*_min_ ≥ 10 and *λ*_max_/*λ*_int_ < 3 were kept.

**Figure 3.**
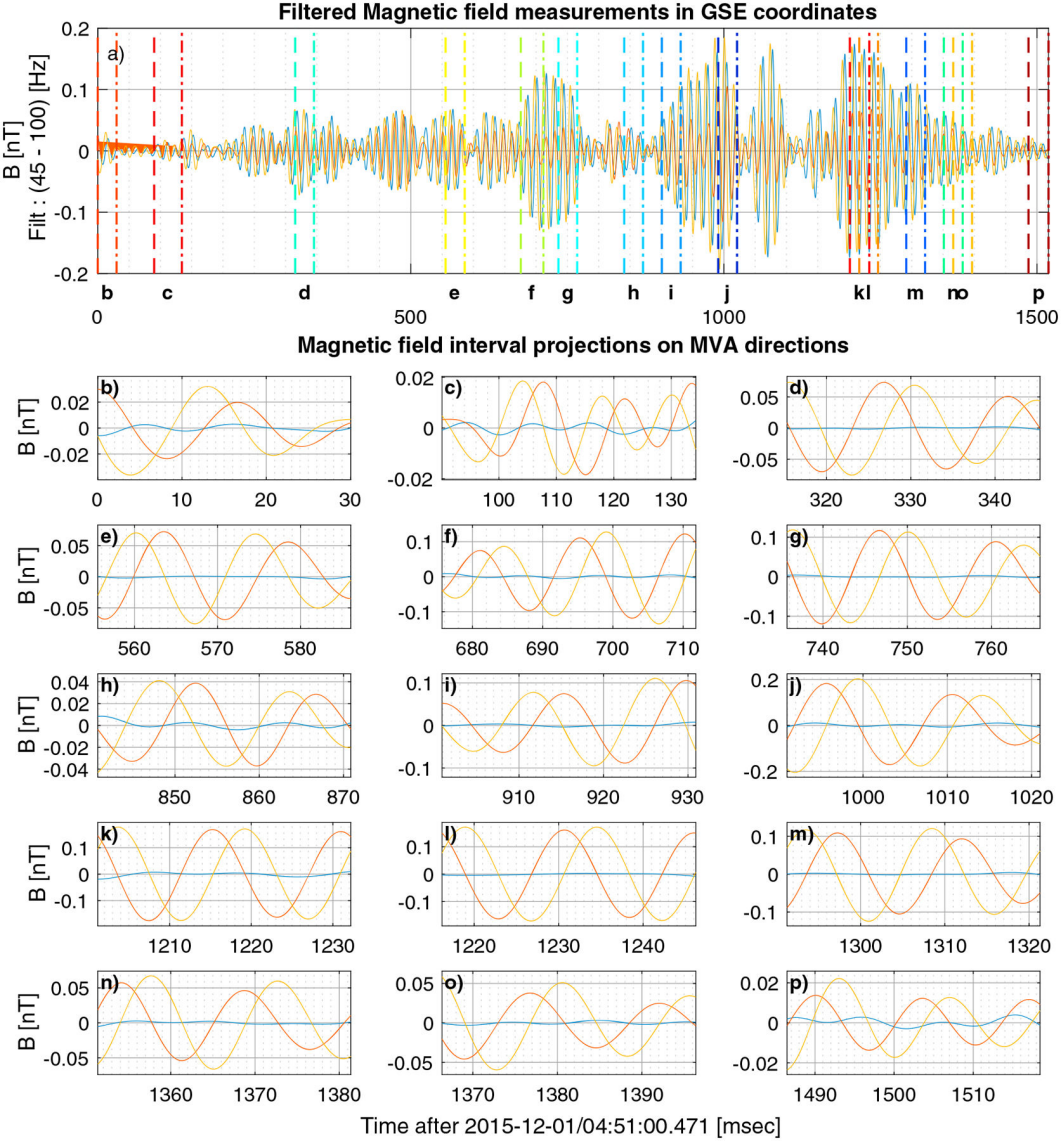
Filtered magnetic field measurements in GSE coordinates (a) for the example emission on 1 December 2015. The magnetic field projections on the MVA directions for each interval (b-p). The minimum, intermediate, and maximum directions are shown in blue, red, and orange, respectively. MVA = minimum variance analysis.

For the LR example shown in Figures 1 and 2, the adaptive minimum variance obtained 16 intervals that satisfied the conditions previously described. The measured and band-pass filtered **B** in GSE coordinates is shown in Figure 3a, where the *x, y*, and *z* coordinates are shown in blue, red, and yellow, respectively. Figures 3b–3p show the magnetic field components in the MVA coordinate basis for that subinterval. The subintervals are shown in Figure 3a as the color-coded dashed (start) and dash-dotted (end) vertical lines. One thing that can be noticed in Figure 3a is that the subintervals do not cover the entire LR interval that was originally identified. One reason for this is that the eigenvalue ratios were not such as to ensure the good quality of the estimates. Another possible reason is that two subintervals, both with good eigenvalue ratios, but not equal, overlapped for a larger part than allowed, which results in the selection of the best of the two. The method attempts to avoid cross contamination between multiple frequencies with different propagation properties but not different modes. All the subintervals may be whistlers, but different frequencies may have different *θ*_*kB*_.

**Figure 4.**
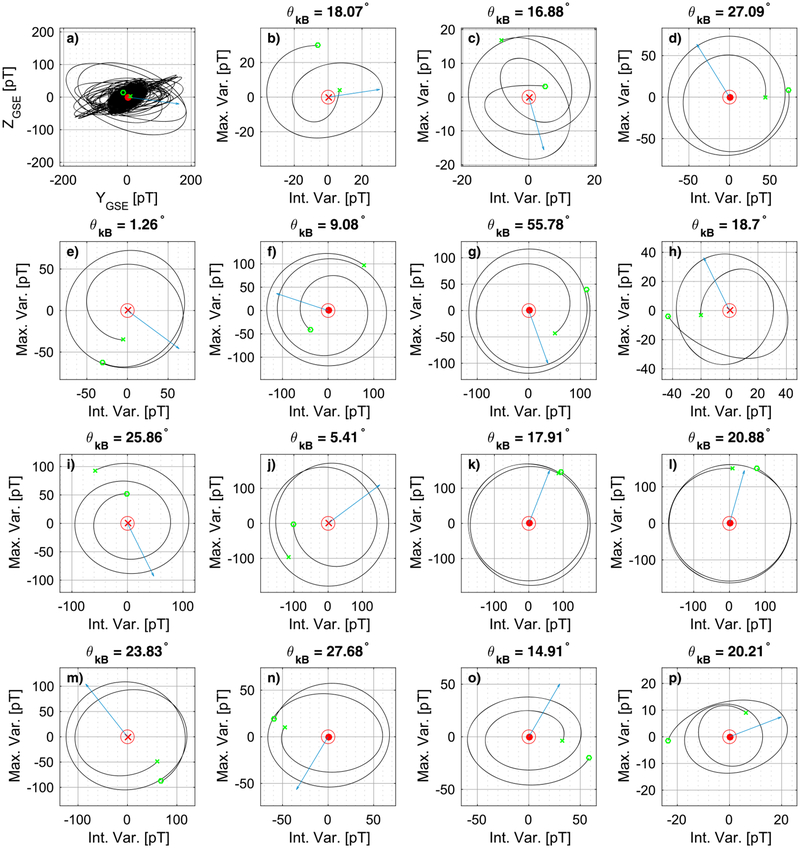
(a-p) Hodograms of the magnetic field maximum and intermediate components for the individual minimum variance analysis intervals of the lion roar example. The blue arrow shows the direction of the background magnetic field (**B**_0_) projected onto the plane of the maximum and intermediate minimum variance analysis direction. The direction of the minimum variance is shown in the origin of the plots with a dot/cross mark.

Figure 4 shows the hodograms of the intervals of Figure 3, and each panel of Figure 4 corresponds to the same panel of Figure 3 marked with the same letter. For example, Figure 4k shows the hodogram of the subinterval of Figure 3k. The starting point of each hodogram is signified by the green circle and the end by the green cross mark. The direction of the minimum variance eigenvector is shown in the center of each panel, where a dot shows that it is directed outside of the paper, while an x mark shows that it is directed inside the paper. The blue arrow shows the direction of **B**_0_ projected onto the plane of the maximum and intermediate MVA directions. Figure 4a shows the hodogram of the entire time series on the MVA coordinates calculated for the entire LR interval. What can be seen is that there are parts where the waves are mostly elliptically polarized, but they appear to change orientation, while there are some parts where they might be circularly polarized. On the other hand, the majority of the subintervals in Figures 4b–4p are almost circularly polarized, with the exception of the subintervals in Figures 4b, 4n, 4o, and 4p that are slightly more elliptical. In the subinterval of Figure 4c there seems to be some rotation of the ellipse in the three windings as well. Finally, the *θ*_*kB*_ angle is calculated for each subinterval and is shown for all subplots of Figure 4. The average *θ*_*kB*_ angle for the 15 subplots is ~ 20°. The majority of the subintervals have a *θ*_*kB*_ angle which is within one standard deviation (i.e., on the interval [7.8–32.7]°).

Calculations for each MVA subinterval shown in Figure 3 are provided in Table 1. The MVA intervals *e, f*, and *j* have much more parallel propagation vector to *B*_0_, while interval *g* propagates in a direction that is more than 45° relative to *B*_0_ and the rest of the MVA intervals have *θ*_*kB*_ ~ 25°. On the other hand *θ*_*kV*_, the angle between the propagation vector and the plasma bulk velocity, is more constant for the majority of the intervals ~ 84° ± 5, with the exception of intervals *b, d*, and *g* (smallest with *θ*_*kV*_ = 67°).

The high sampling frequency of **B** allowed for high-frequency waves to be observed. This leads to having MVA subintervals that last ^⇀^100 ms. On the other hand, the sampling of **B**_0_, *n*, and **V**_*b*_ is considerably smaller. This is why these quantities were resampled with the sampling rate of **B** using linear interpolation for the points that lie between two actual samples of the three quantities.

In order to obtain the wavenumber for each of the MVA intervals, we follow a similar procedure to that outlined in Wilson et al. (2013). The cold plasma index of refraction for oblique whistler waves satisfying *ω*_rf_^2^ ≪ *ω*_*pe*_^2^ and *ω*_*ce*_ ≪ *ω*_*pe*_ is given by

(1) $$n^{2}=\frac{k^{2}c^{2}}{\omega_{\text{rf}}{}^{2}}=\frac{\omega_{pe}{}^{2}}{\omega_{\text{rf}}\left(\omega_{ce}\text{cos}\left(\theta_{kB}\right)-\omega_{\text{rf}}\right)}$$

where *ω*_*pe*_ is the electron plasma frequency, *ω*_*ce*_ is the electron cyclotron frequency, and *ω*_rf_ is the frequency of the wave in the plasma rest frame. Applying a Doppler shift (*ω*_*sc*_ = *ω*_rf_ − **k** · **V**_*b*_) to equation (1) (where *ω*_*sc*_ is the frequency observed in the rest frame and **V**_*b*_ the plasma bulk velocity) leads to the third-order polynomial

(2) $${\bar{V}}_{b}{\hat{k}}^{3}+\left(\text{cos}\left(\theta_{kB}\right)-{\bar{\omega }}_{sc}\right){\hat{k}}^{2}+{\bar{V}}_{b}\hat{k}-{\bar{\omega }}_{sc}=0$$

where $\hat{k}=kc/\omega_{pe}$, ${\bar{V}}_{b}=V_{b}\text{cos}\left(\theta_{kV}\right)/V_{Ae}$, ${\bar{\omega }}_{sc}=\omega_{sc}/\omega_{ce}$, *V*_*Ae*_ = *B*_0_/(*μ*_0_*n*_*e*_*m*_*e*_)^1/2^, *μ*_0_ is the free space permeability, *n*_*e*_ is the electron density, and *m*_*e*_ is the electron mass.

The real part of the roots of equation (2) provides three solutions for *ω*_rf_. Since we are interested in high-frequency whistler waves, the valid solutions are the ones where *ω*_rf_ > *ω*_LH_, where *ω*_LH_ = [(*ω*_*ci*_*ω*_*ce*_)^−1^ + *ω*_*pi*_^−2^]^−1/2^, $\omega_{pi}=\sqrt{n_{i}e^{2}/\left(m_{i}\epsilon_{0}\right)}$ is the ion plasma frequency, *n*_*i*_ is the density of ions, *B* is the magnetic field magnitude, *e* is the elementary charge, *c* is the speed of light, and *m*_*i*_ and *m*_*e*_ are the mass of the ion and electron, respectively.

To determine the properties of the waves in section 3, the probability density function (PDF) was fitted to the data, using kernel density estimation defined by

(3) $$f(x)=\frac{1}{nh}\overset{n}{\underset{i=1}{\sum }}K\left(\frac{x-x_{i}}{h}\right)$$

where *n* is the number of samples, *x*_*i*_ is each individual sample, *K* is the kernel function, and *h =* 1.06*σn*^−1/5^ is generally used as a rule of thumb, with *σ* being the estimated standard deviation of the sample. For a more detailed explanation of the kernel density estimation, see Silverman (1986). Experimentally determining the PDF of the samples allows then to estimate the cumulative density function (*F*(*x*)) by integration, as well as obtain the expected value defined by

(4) $$E(x)=\int_{-\infty }^{+\infty }xf(x)\text{d}x$$

The cumulative density function (CDF) can then be used to determine the probability $P\left(x<x_{i}\right)=F\left(x_{i}\right)=\int_{0}^{x_{i}}f(x)dx$ or inside an interval A, by finding the area of the PDF in that interval.

**Figure 5.**
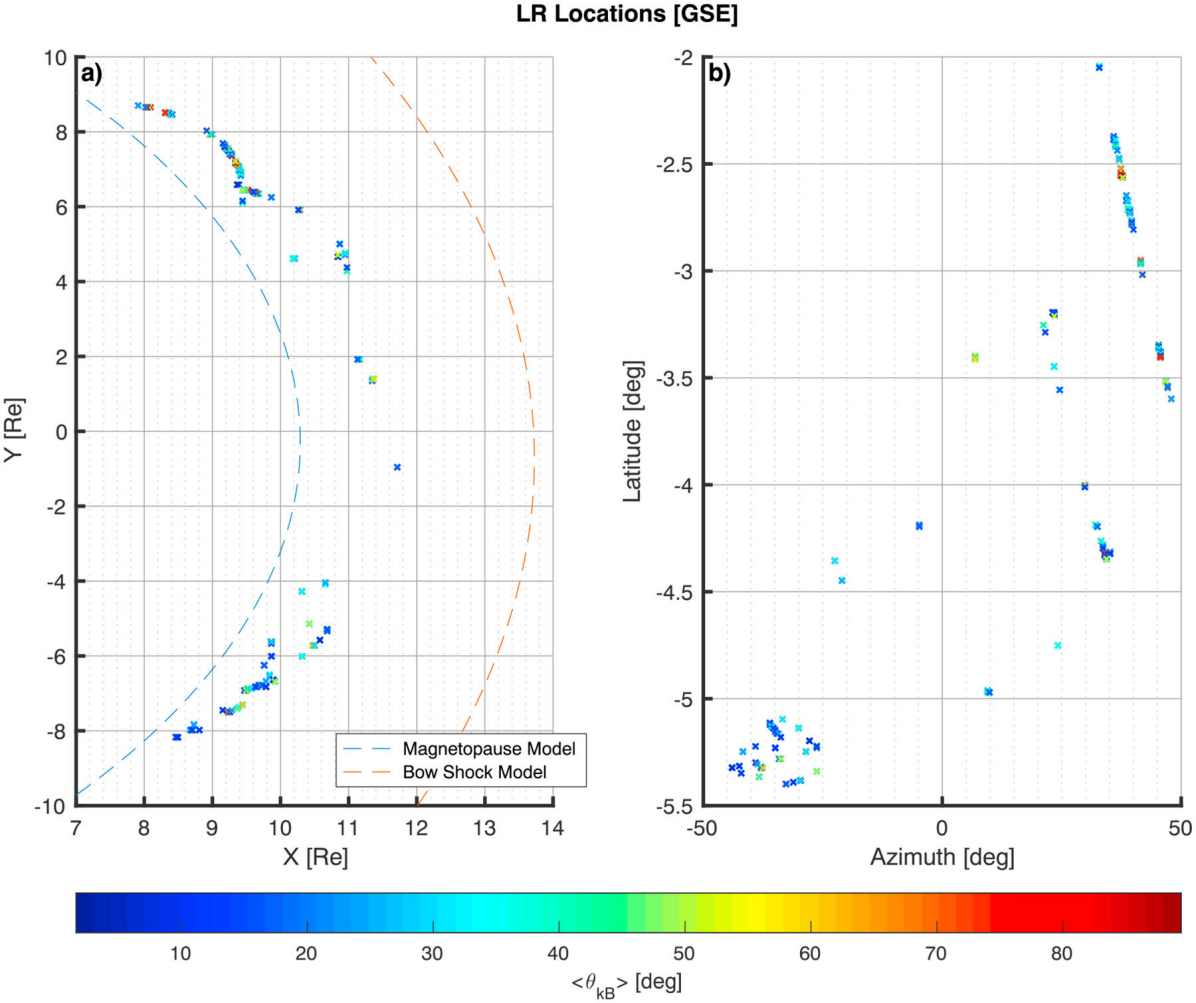
Locations of observed LR emissions by the Magnetospheric Multiscale 3 on 18 different dates from 16 October 2015 to 13 January 2016. The 1,738 locations shown are the ones where the minimum variance analysis eigenvalues were considered good. The *x-y* positions of the spacecraft at the time of observation are shown in panel a, along with a model magnetopause (blue dashed line) and model bow shock (red dashed line). In panel b the latitude versus the azimuth angle of the position of the observed LR is shown. The points in both panels follow the same color coding for the angle between the background magnetic field and the average propagation direction of each LR interval observed $\left(\langle \theta_{kB_{0}}\rangle \right)$. LR = lion roar.

The locations where emissions were detected are shown in Figure 5. The magnetopause and bow shock models are also shown in Figure 5. A wide range of the magnetosheath *x-y* plane was sampled, but due to the trajectories of the spacecraft, the sample range of latitudes is not as broad. The location of all the LRs that was observed being closer to the magnetopause is probably due to the majority of the timing when the satellites were in burst mode operation that was set to coincide closer to magnetopause crossings.

## 3. Statistical Results

**Figure 6.**
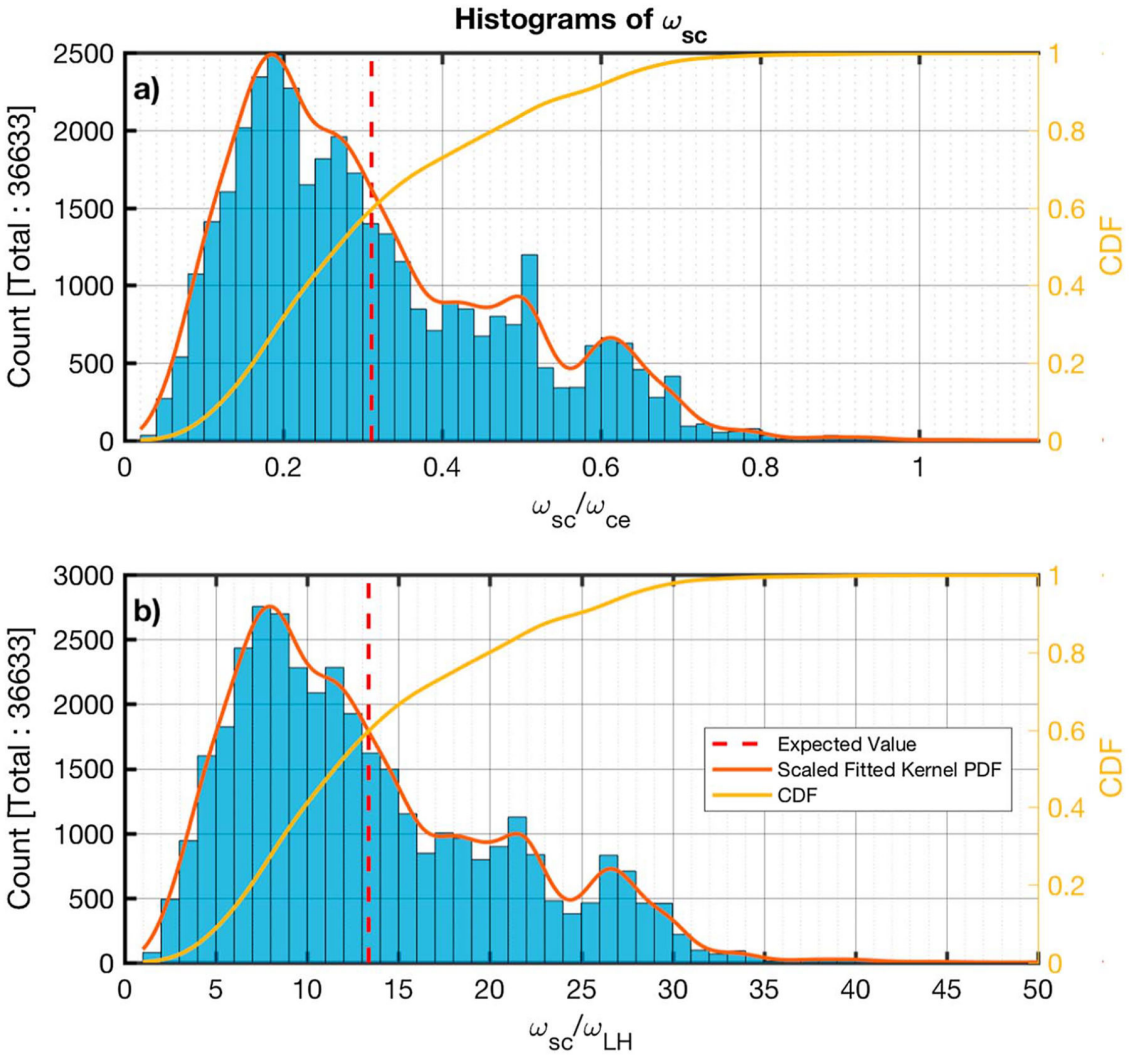
Histograms of the ratios of the spacecraft frame frequency peak (*ω*_*sc*_) identified to the electron cyclotron (panel a) and the lower hybrid frequency (panel b), for each minimum variance analysis subinterval. The estimated scaled fitted PDF and the CDF are also shown in red and yellow, respectively. The calculated average is shown by the dashed vertical red line. CDF = cumulative density function; PDF = probability density function.

**Figure 7.**
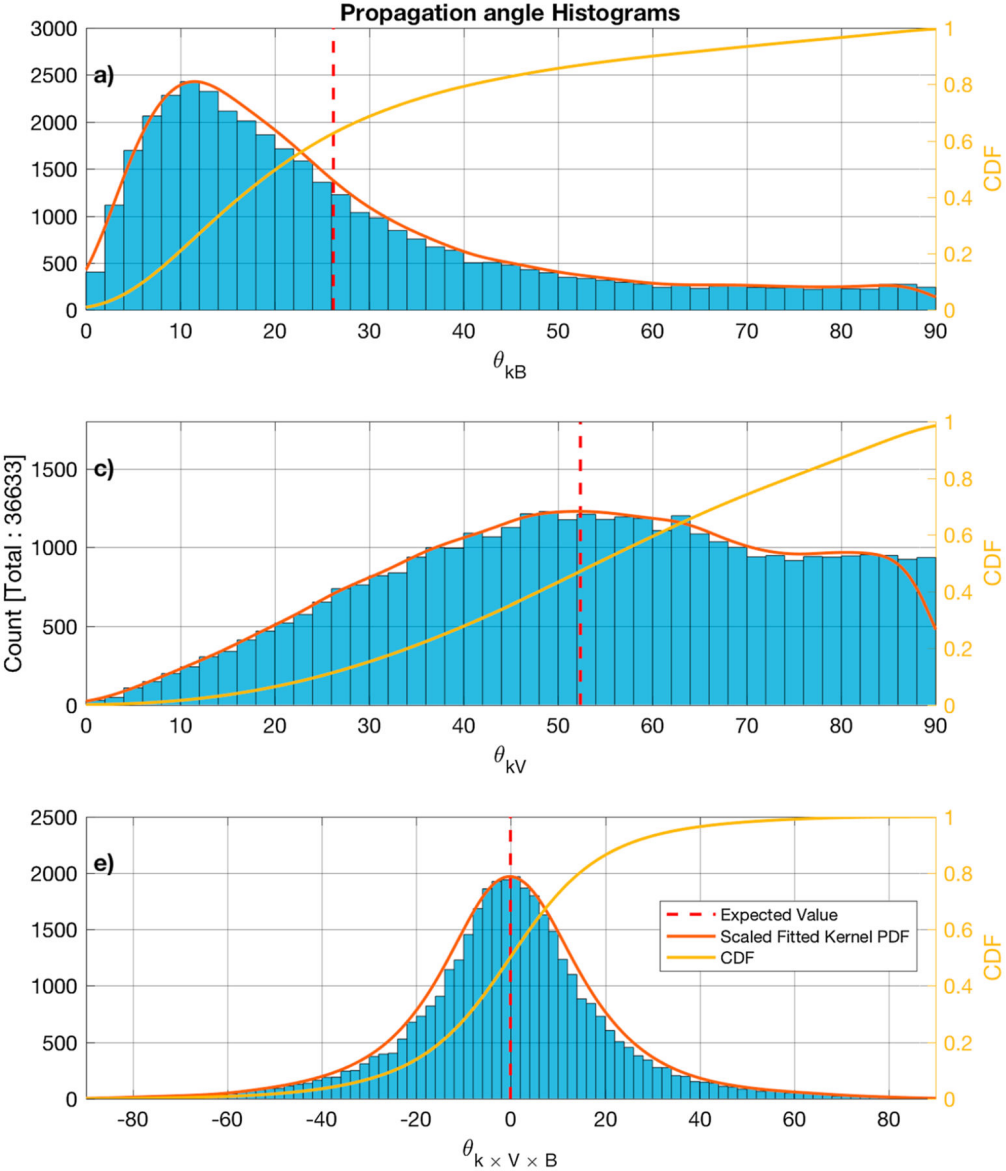
Histograms of the propagation vector angle with the background magnetic field (panel a), the plasma bulk flow (panel b), and the latitude from the **V** × **B** plane (panel c). The estimated scaled fitted PDF and the CDF are also shown in red and yellow, respectively. The calculated average is shown by the dashed vertical red line. CDF = cumulative density function; PDF = probability density function.

**Figure 8.**
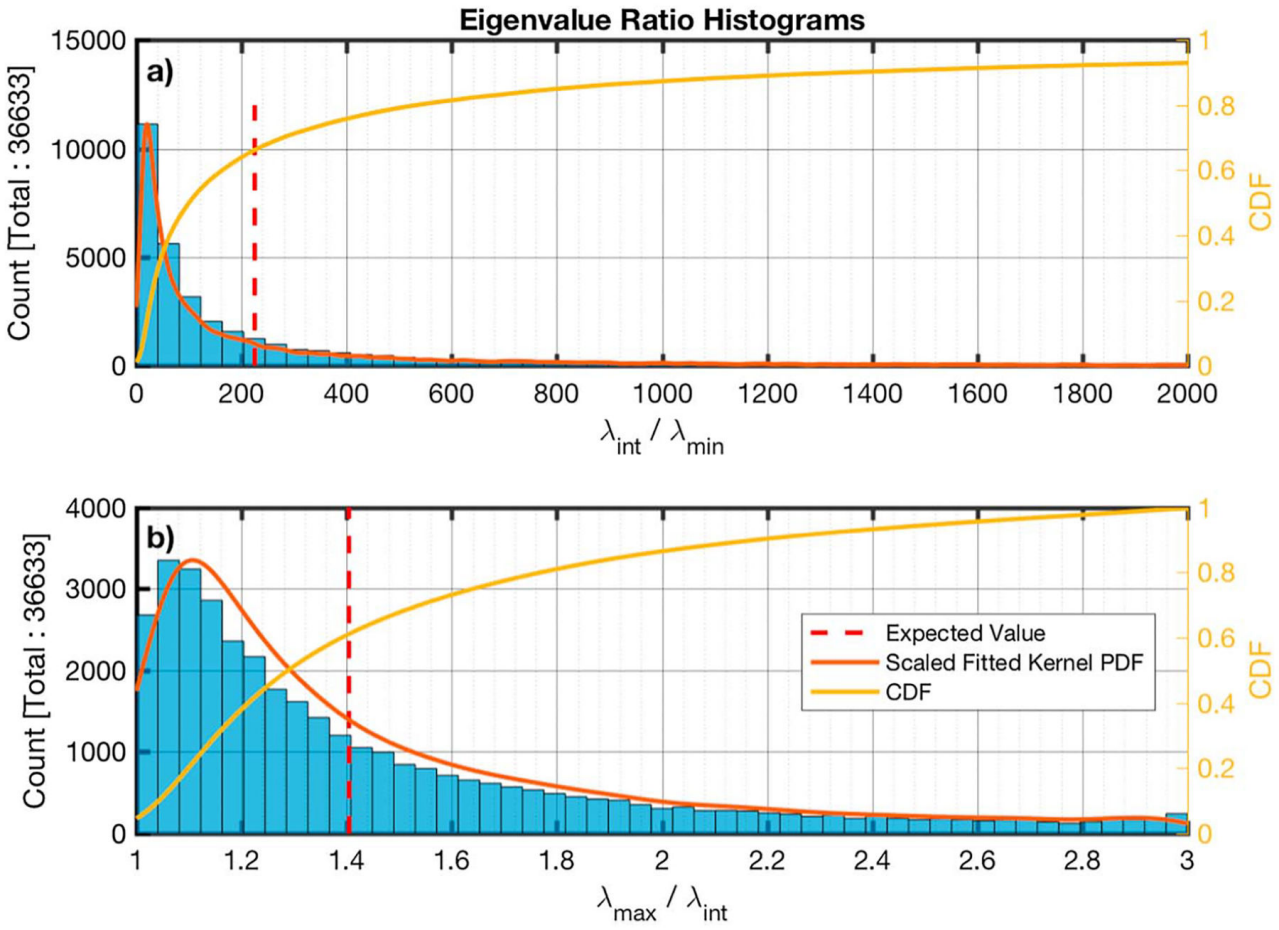
Histograms of the eigenvalue ratios of the intermediate to minimum (panel a) and the maximum to intermediate (panel b) minimum variance analysis components. The estimated scaled fitted PDF and the CDF are also shown in red and yellow, respectively. The calculated average is shown by the dashed vertical red line. CDF = cumulative density function; PDF = probability density function.

**Figure 9.**
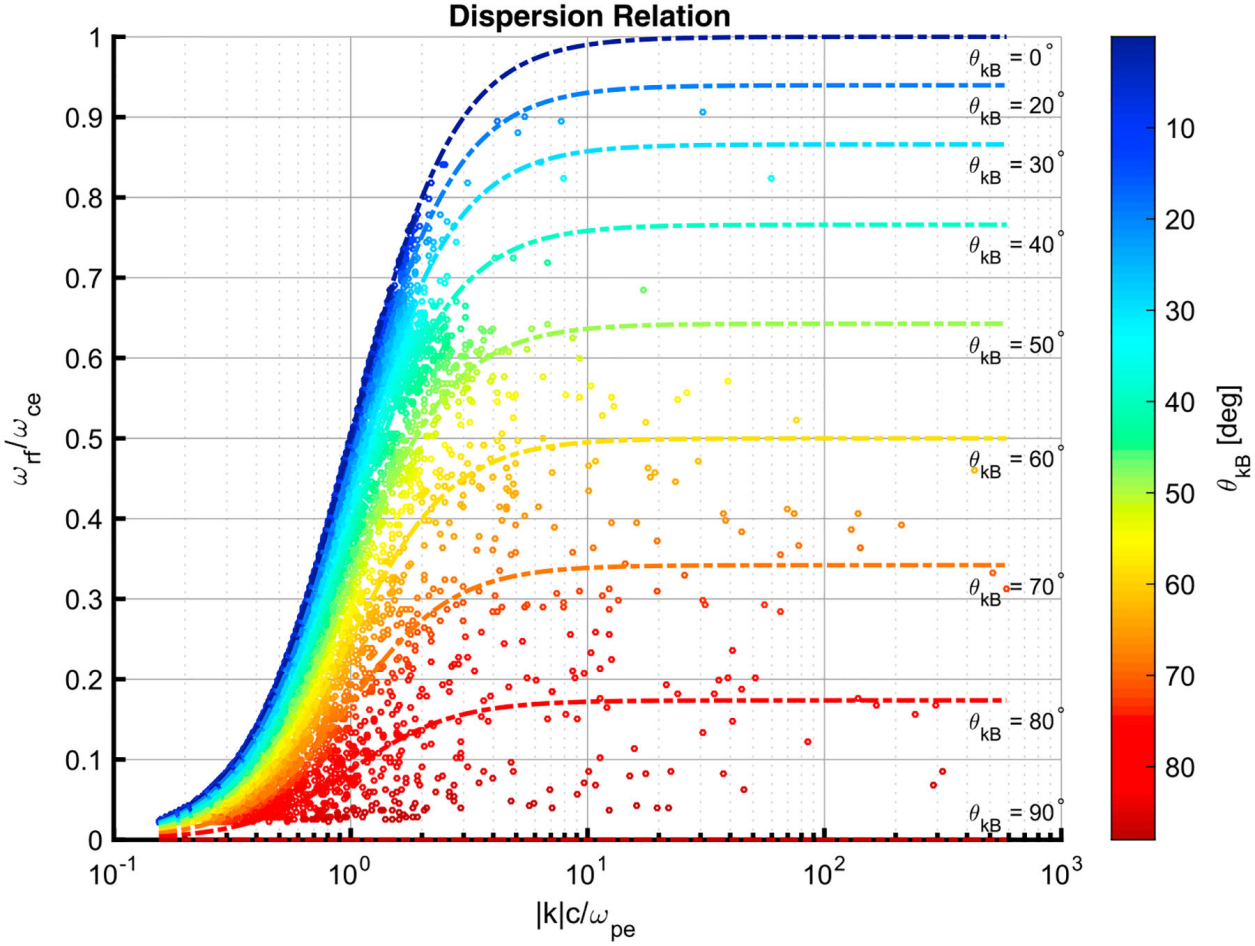
Dispersion relation for the 30,636 minimum variance analysis intervals where *ω*_rf_ > *ω*_LH_. The circles are the points of the individual minimum variance analysis subintervals. The dashed lines show the calculated dispersion for a given *θ*_*kB*_ angle.

**Figure 10.**
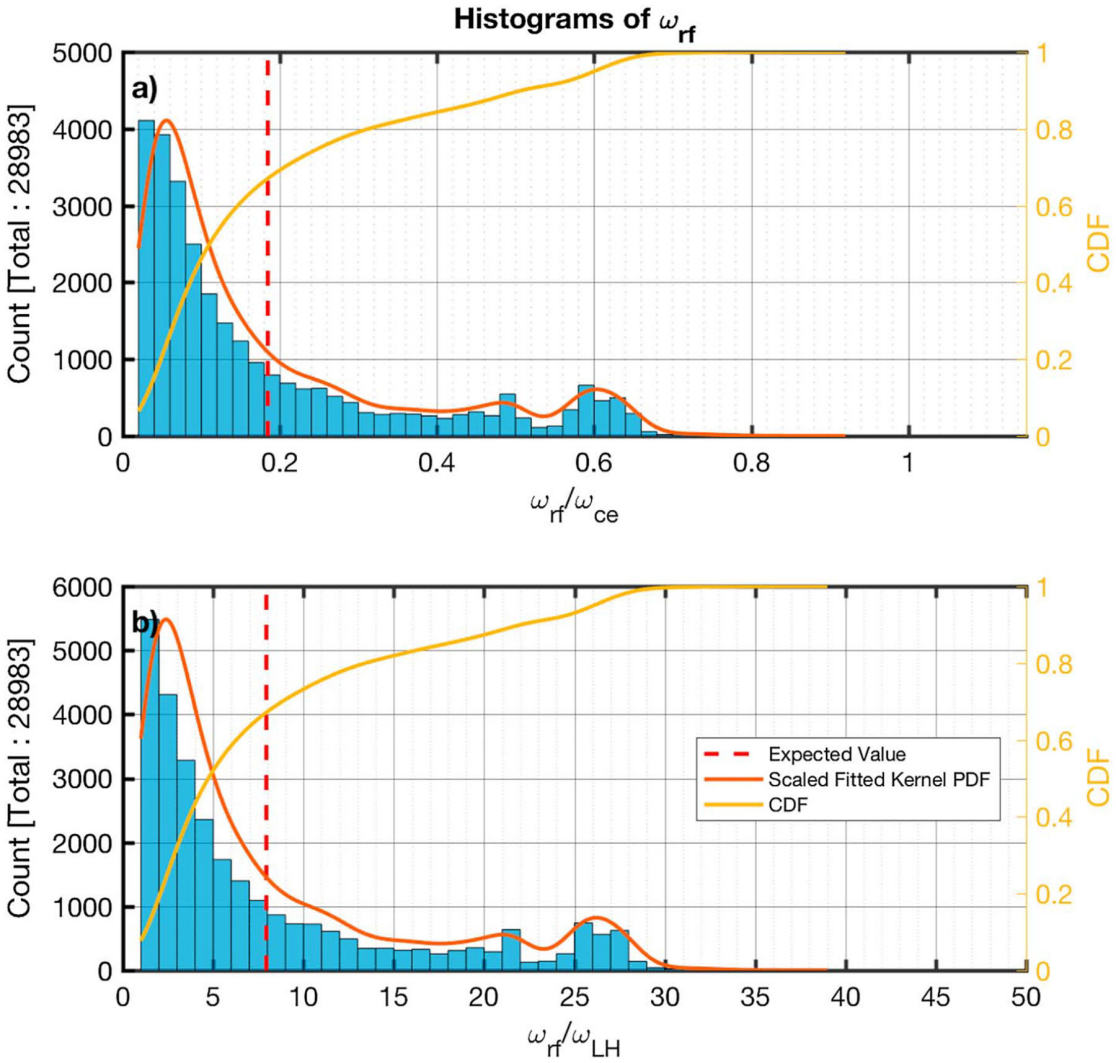
Histograms of the ratios of the rest frame frequency peak (*ω*_rf_) identified to the electron cyclotron (panel *a*) and the lower hybrid frequency (panel *b*), for each minimum variance analysis subinterval. The estimated scaled fitted PDF and the CDF are also shown in red and yellow, respectively. The calculated average is shown by the dashed vertical red line. CDF = cumulative density function; PDF = probability density function.

**Figure 11.**
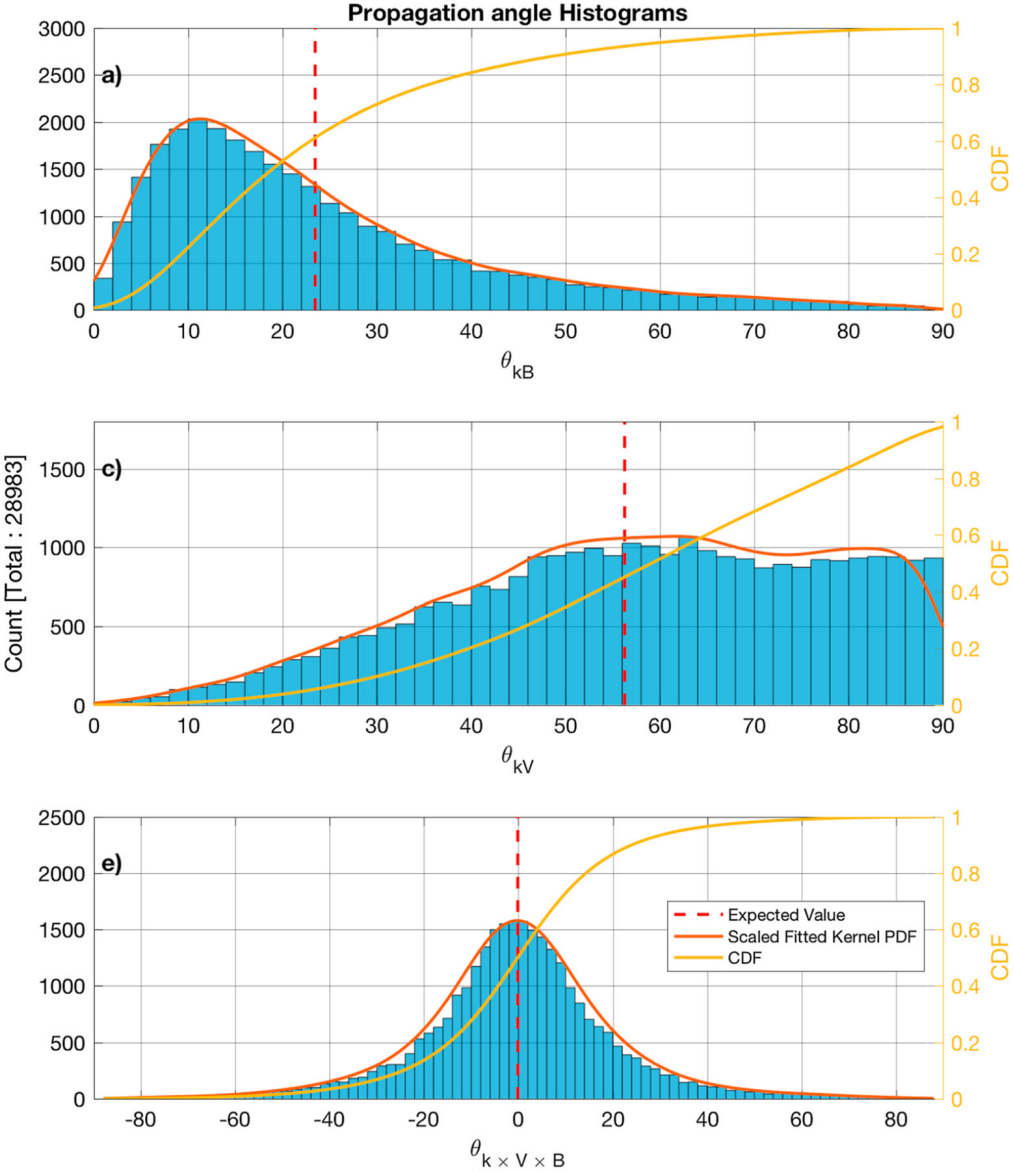
Histograms of the propagation vector angle with the background magnetic field (panel a), the plasma bulk flow (panel b), and the latitude from the **V** × **B** plane (panel c) for the minimum variance analysis subintervals for which *ω*_rf_ > *ω*_LH_. The estimated scaled fitted PDF and the CDF are also shown in red and yellow, respectively. The calculated average is shown by the dashed vertical red line. CDF = cumulative density function; PDF = probability density function.

**Figure 12.**
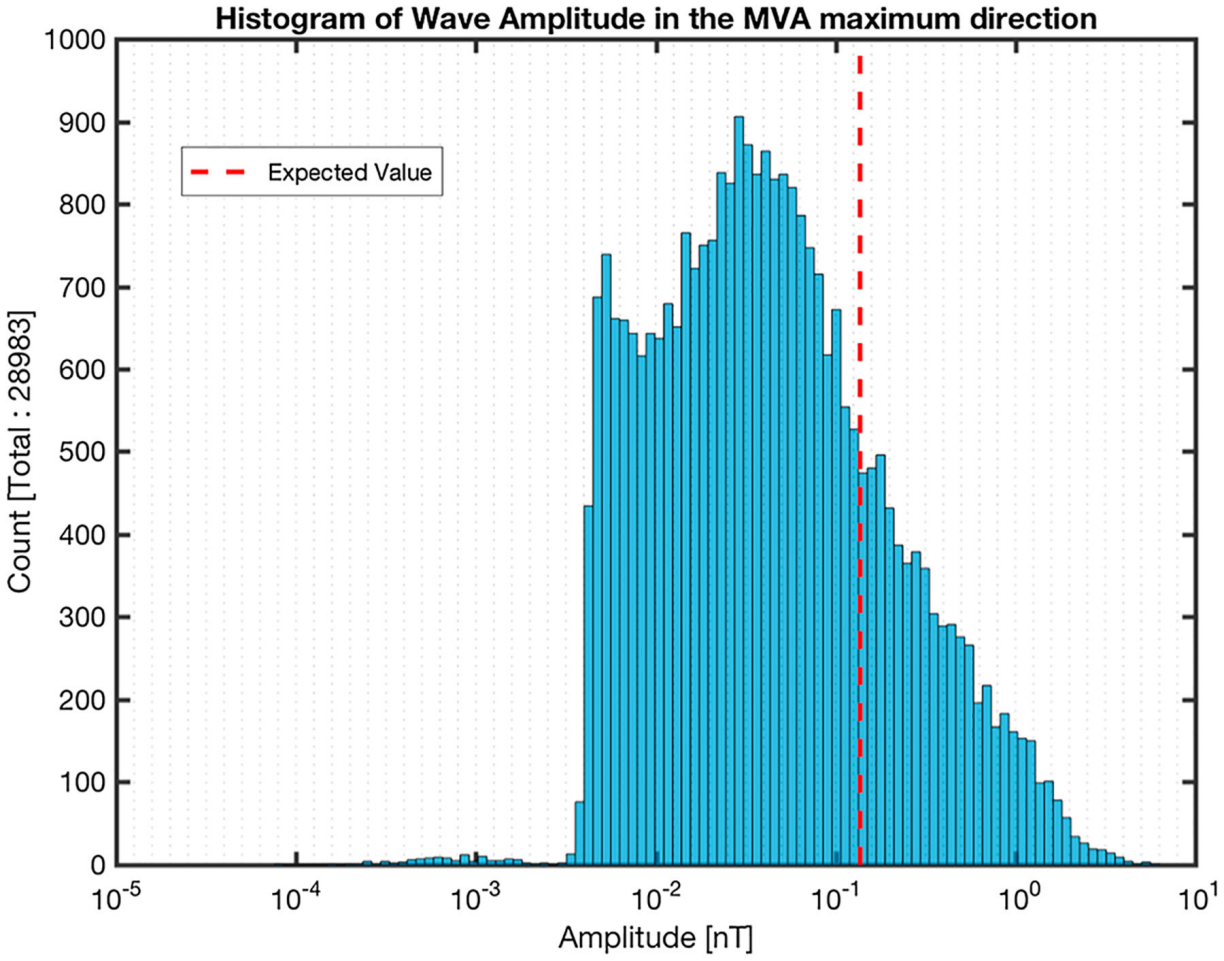
Histogram of the amplitude in the maximum MVA coordinate of the MVA subintervals where *ω*_rf_ > *ω*_LH_. The expected value obtained by the probability density function fitted to the data is shown. The fitted probability density function is not shown because it was not estimated for the logarithm of the data. MVA = minimum variance analysis.

**Figure 13.**
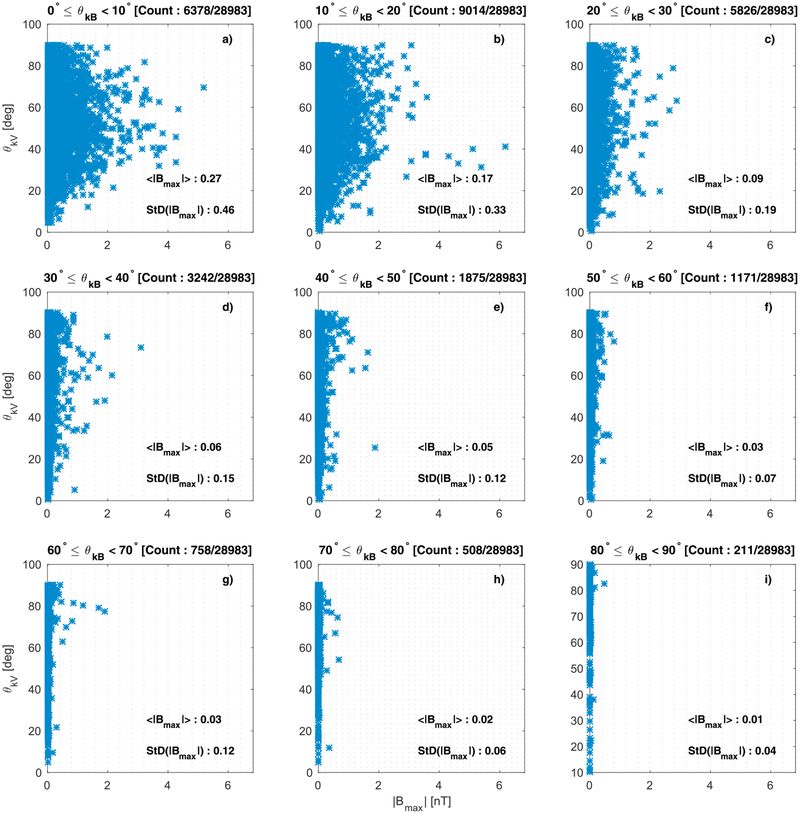
(a-i) Plots of *θ*_*kV*_ against the amplitude in the maximum minimum variance analysis coordinate of the minimum variance analysis subintervals where *ω*_rf_ > *ω*_LH_. In each panel emissions with the *θ*_*kB*_ range are shown. The mean (< |**B**_max_| >) and the standard deviation (StD(|**B**_max_|)) for each interval of *θ*_*kB*_ are also shown.

The MVA analysis yielded 39,709 subintervals from a total of 1,738 LR intervals identified, using data from 18 different dates between 16 October 2015 and 13 January 2016. From these MVA subintervals, 2,115 were excluded from the study, because the particle density was measured to be ≥ 75 cm^−3^ which is a region where the fast plasma investigation instrument is inaccurate due to saturation effects. About 961 MVA intervals were removed because measurements of the ion and/or electron distributions were not available. The wave properties are summarized in Figures 6–13.

Figures 6a and 6b show histograms of the ratios of the peak frequency over the electron cyclotron (*ω*_*ce*_ = *eB/m*_*e*_*c*) and the lower hybrid (*ω*_LH_ = ((*ω*_*ci*_*ω*_*ce*_)^−1^ + *ω*_*pi*_^−2^)^−1/2^) frequency, respectively. The estimated PDF is plotted on top of the histograms along with the CDF in panel b of both figures.

Figures 7a–7c show the distribution of the MVA subintervals *θ*_*kB*_*, θ*_*kV*_, and *θ*_*k×V×B*_ angles, where *θ*_*k×V×B*_ = 90° − *cos*^−1^ (**k** · (**V**_*b*_ × **B**_0_)). The MVA eigenvalue ratio of the intermediate to minimum in Figure 8a and the maximum to intermediate directions is shown in Figure 8b.

For each of the MVA subintervals, the peak frequency of the emissions in the spacecraft frame, along with plasma measurements, was used with equation (2) to all the MVA results. In total, 30,636 of the MVA intervals had *ω*_rf_ > *ω*_LH_. Figure 9 shows the plot of the normalized peak frequency (*ω*_rf_/*ω*_*ce*_) against the normalized wave vector magnitude ($\left|\hat{k}\right|$).

The histograms for the ratio of the rest frame peak frequency to the electron cyclotron and lower hybrid frequency are shown in Figures 10a and 10b, respectively, and the formatting is the same as the previous histogram figures.

The histograms of the angles *θ*_*kB*_*, θ*_*kV*_, and *θ*_*k×V×B*_ for the 30,636 MVA subintervals for which the rest frame frequency adheres to the condition *ω*_rf_ > *ω*_LH_ are shown in Figures 11a–11c, respectively.

A histogram of the magnetic field amplitude in the maximum MVA coordinate (**B**_max_) is shown in Figure 12 for the subintervals where equation (2) had a valid solution. In this case the PDF was estimated for the actual data and not the logarithm of the data, which is shown in the plot for convenience. The amplitude of the same subintervals is plotted relative to the *θ*_*kV*_ angle in Figure 13. Figures 13a–13i show the plots of *θ*_*kV*_ against the maximum amplitude of the same subintervals from Figure 12 with each panel showing the subintervals within the *θ*_*kB*_ as indicated in the figures.

**Figure 14.**
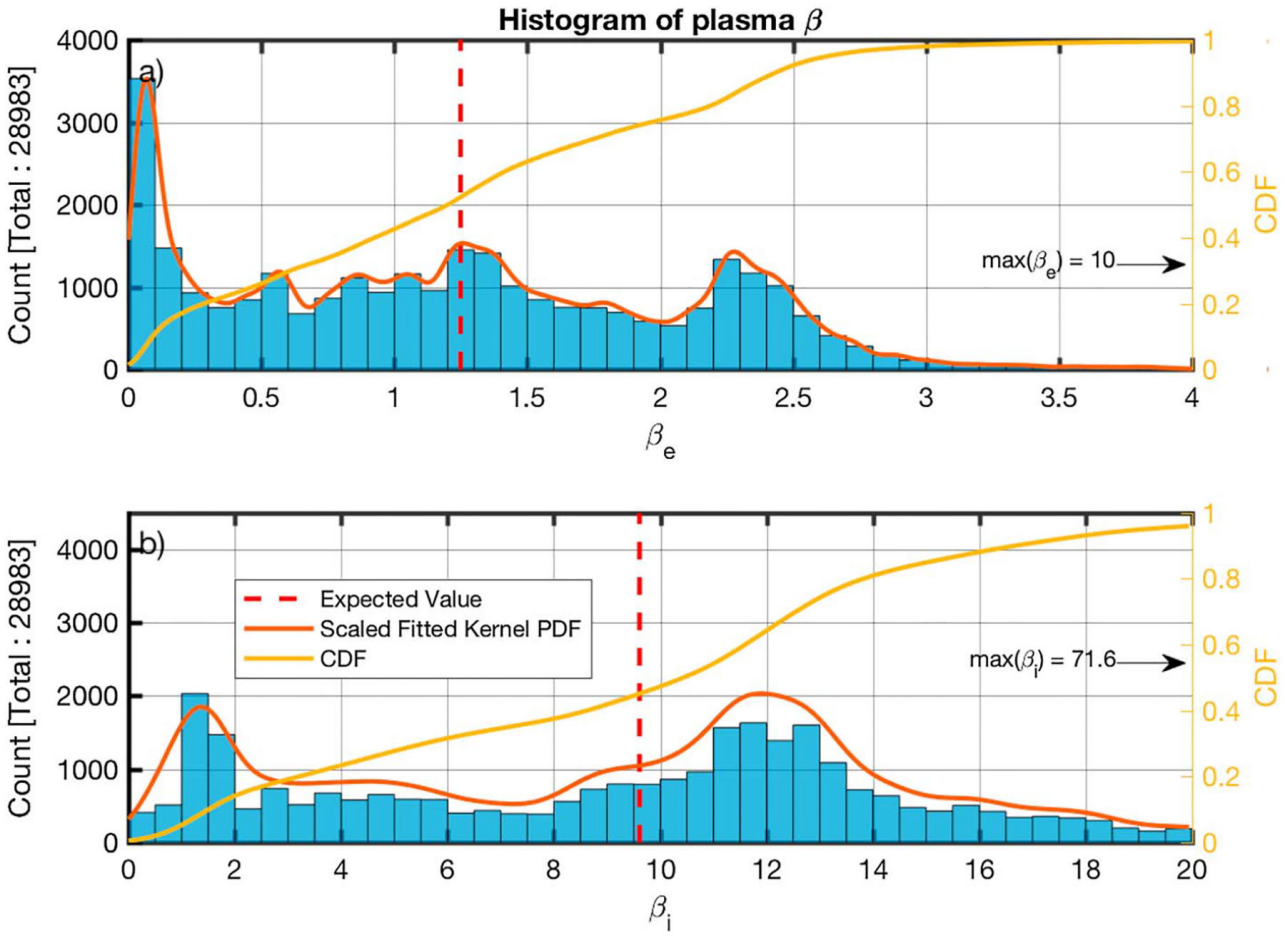
Histograms of the calculated plasma beta for the electrons (panel a) and ions (panel b) for the minimum variance analysis subintervals for which *ω*_rf_ > *ω*_LH_. The estimated scaled fitted PDF and the CDF are also shown in red and yellow, respectively. The calculated average is shown by the dashed vertical red line. CDF = cumulative density function; PDF = probability density function.

The histograms of the plasma beta for the electrons and the ions for the 28,983 MVA results for which *ω*_rf_ > *ω*_LH_ is true are shown in Figures 14a and 14b. In both figures the *x* axis has been limited and the maximum value for each case is indicated. In the case of electrons, the number of subintervals where *β*_*e*_ > 7 is 16 and in the case of the ions, there are 1,289 subintervals with *β*_*i*_ > 20.

## 4. Discussion and Conclusions

We have examined the properties of circularly polarized electromagnetic waves from 36,633 subintervals of 1,738 intervals of magnetic field measurements from MMS that were identified as lion roar emissions. The 1,738 intervals were automatically identified based on the magnetic field measurements with the main constraint that they are primarily transverse waves. The frequency band of the lion roars in each interval was manually identified based on the power spectrum of the magnetic field of the intervals. The intervals were then submitted to an automatic adaptive interval algorithm that uses MVA to identify appropriate subintervals. From all the MVA subintervals, only the ones that were circularly polarized and had adequately large eigenvalue ratios, to ensure high accuracy in the estimation of **k**, were kept. Using the cold plasma index of refraction for oblique whistler waves along with the Doppler shift, we obtained *ω*_rf_ and |*k*| for each MVA subinterval. From the original subintervals, 28,983 satisfied *ω*_rf_ > *ω*_LH_ and they were further examined. No obvious correlation could be found between the coefficients of equation (2) and the lack of a solution that satisfied *ω*_rf_ > *ω*_LH_ for the other 7,650.

From the dispersion relation plot in Figure 9, we can see that the frequency-wavenumber plane has been well sampled for all propagation directions up to ~ 2.4 k c/*ω*_*pe*_. For wavenumbers > 2.4 k c/*ω*_*pe*_ there are more samples for waves that propagate at *θ*_*kB*_ > 40°.

The plasma beta for the ions (Figure 14b) has *E*(*β*_*i*_) ~ 9.6, and the histogram peaks at around 1.4 and 12. For the electrons (Figure 14a), *E*(*β*_*e*_) ~ 1.2 and 98% of measurements are < 4.

The majority of the emissions (99.8%) have *ω*_*sc*_ < *ω*_*ce*_ (Figure 6a) and (99.7%) *ω*_LH_ < *ω*_*sc*_ < 48*ω*_LH_ (Figure 6b). Based on the PDF of *ω*_*sc*_*/ω*_*ce*_ and *ω*_*sc*_/*ω*_LH_, the distribution has three peaks, the first one being substantially larger, at ~ 0.19*ω*_*ce*_ (~ 8.1*ω*_LH_), ~ 0.49*ω*_*ce*_ (~ 21*ω*_LH_), and ~ 0.61*ω*_*ce*_ (~ 27*ω*_LH_). The average (expected value shown in equation (4) frequency is 0.3*ω*_*ce*_ and 13.4*ω*_LH_. In the plasma rest frame, we observe that the distributions have been shifted to lower frequencies and the majority of the subintervals (98%) have *ω*_rf_ < 0.72*ω*_*ce*_ (Figure 10a) and (92%) *ω*_LH_ < *ω*_rf_ < 30*ω*_LH_ (Figure 10b). The shape of the distributions has also changed significantly. The peak at ~ 0.61 *ω*_*ce*_ (~ 27*ω*_LH_) still appears with a similar magnitude, but the distribution left of this point now resembles more an exponential decay, with a peak at ~ 0.06*ω*_*ce*_ (~ 2.4*ω*_LH_). The average value of *ω*_rf_ is ~ 0.18*ω*_*ce*_ (7.9*ω*_LH_). The average frequency in the rest frame is about half of that in the spacecraft frame.

The minor differences that are seen between the pairs of Figures 7a and 11a, 7b and 11b, and 7c and 11c are due to the exclusion of some subintervals because no valid solutions could be found for equation (2). Comparing the histograms of the *θ*_*kB*_ angle between the subintervals in the spacecraft frame (Figure 7a) and the doppler shifted results (Figure 11a), the subintervals with *θ*_*kB*_ ~ 90° are not present and the transition from 60° to 90° has a negative slope, while in Figure 7a it appears to be more flat. Similarly, the histogram of the *θ*_*kV*_ angle of the subintervals in the spacecraft frame (Figure 7b) appears to peak ~ 50°, which is not observed in the Doppler shifted results (Figure 11b). The shape of the histograms for the *θ*_*k×V×B*_ (Figures 7c and 11c) is similar in both cases.

From the estimated PDFs for all the MVA subintervals (Figures 7a–7c), the expected values are *E*(*θ*_*kB*_) = 26°, *E*(*θ*_*kV*_) = 52°, and *E*(*θ*_*k×V×B*_) ~ 0°, while 81% of the samples have *θ*_*kB*_ < 45°, *θ*_*kV*_ > 32°, and −26° < *θ*_*k×V×B*_ < 26°. When considering the valid Doppler shifted-only subintervals, the expected values are *E*(*θ*_*kB*_) = 23°, *E*(*θ*_*kV*_) = 56°, and *E*(*θ*_*k×V×B*_) ~ 0° and 81% of the samples have *θ*_*kB*_ < 38°, *θ*_*kV*_ > 37°, and −26° < *θ*_*k×V×B*_ < 26°.

The majority of LRs propagate obliquely relative to the plasma bulk flow and are more likely to be observed to propagate close to parallel to the background magnetic field. The average value of *θ*_*k×V×B*_ ~ 0° could indicate that the free energy source for the waves is mainly linked to the magnetic field and the plasma bulk flow. The peak that was observed at *θ*_*kV*_ ~ 50° is consistent with the results of Wilson et al. (2013), where they argued that the lower sampling frequencies were the reason that waves with lower *θ*_*kV*_ were not observed due to a Doppler shift above the Nyquist frequency. On the other hand, the data used in this study were sampled at much higher frequency and the same phenomenon is observed.

The majority of the studies have observed few cases of LRs with *θ*_*kV*_ < 45°. Wilson et al. (2013) reported no observations of whistler waves with such angles, Moullard et al. (1998) reported an average 70°, and from the examples of Zhang et al. (1998) we found two cases with *θ*_*kV*_ < 45° from the examples presented in the paper. In this study ~ 35% of the MVA subintervals have *θ*_*kV*_ < 45° and ~ 27% of the Doppler shifted MVA subintervals. The difference results from an inability to calculate the Doppler shift for all MVA subintervals, because there was no solution that solved equation (2) and satisfied the conditions previously mentioned.

Looking at Figures 6a and 6b, and 10a and 10b, it can be seen that the shapes of the distributions of *ω/ω*_*ce*_ and *ω*/*ω*_LH_ both in the spacecraft and the plasma rest frame are similar and the scaling between them is $\sim \sqrt{m_{i}/m_{e}}$. This is because the ion plasma frequency of all measurements is very high. This makes the lower hybrid frequency dependant upon *ω*_*ci*_ and *ω*_*ce*_. More specifically, (*ω*_*ce*_/*ω*_LH_)^2^ ~ *ω*_*ce*_^2^*/*(*ω*_*ce*_*ω*_*ci*_) = *m*_*i*_*/m*_*e*_. Assuming that *n*_*e*_ ~ *n*_*i*_, then *ω*_*pe*_ > *ω*_*pi*_, and so the condition for the waves satisfies *ω*_*ce*_ ≪ *ω*_*pe*_, required by equation (1).

The expected value for the maximum MVA component peak amplitude of the emissions was found to be ~ 0.14 nT, while 77% of the samples have an amplitude < 1 nT. The maximum amplitude found was ~ 6.2 nT. Based on Figures 13a–13i, the largest amplitude emissions were observed for smaller *θ*_*kB*_ angles. For angles *θ*_*kB*_ < 60°, it appears that the amplitudes have a larger mean and standard deviation than in the cases where *θ*_*kB*_ > 60°. Finally, for *θ*_*kB*_ < 80, the amplitude has a smaller range for smaller angles of *θ*_kV_.

The source of LRs in the magnetosheath is most likely a temperature anisotropy of the halo electrons. This anisotropy can in some cases be related to mirror modes, often observed in the magnetosheath. Lengyel-Frey et al. (1994) have calculated the energies for the resonant electrons for each of the cases of Landau damping, cyclotron resonance, and anomalous cyclotron resonance due to the interaction with whistler waves observed at interplanetary shocks. The lower-energy electrons experience Landau interactions, and the higher-energy electrons experience cyclotron interactions. LRs in the magnetosheath will affect the electron distribution similarly since they are oblique and observed at a high range of frequencies. Landau damping will result in a more oblate electron velocity distributions in the direction of the background magnetic field. Cyclotron interactions can cause a temperature anisotropy in the halo electrons. If the interaction results in damping of the wave, then it will increase the temperature in the perpendicular direction relative to the background magnetic field (Brice, 1964). The interaction between the waves and the electrons can lead to a distribution different from the one that generated the waves (Chang et al., 2013; Hughes et al., 2014).

As LRs propagate from the bow shock toward the magnetopause, they play an important role in the regulation of the halo electron anisotropies in the magnetosheath. They also seem to be closely related to mirror mode structures and the regulation of the temperature distribution of the trapped electrons in these structures (Breuillard et al., 2017). Quasi-linear and nonlinear particle-wave interactions could lean to untrapping electrons from the mirror mode. The sampling rate and the quality of the MMS instruments could offer better insight on the mechanisms that generate LRs and how they affect the plasma as they propagate.

Finally, whistler mode waves have been observed in many different regions of the heliosphere, such as magnetic clouds (Moullard et al., 2001) and planetary atmospheres (Hughes et al., 2014), and they are closely linked to collisionless shocks, planetary, and interplanetary (Gary & Mellott, 1985; Lengyel-Frey et al., 1994; Walker et al., 1999). It is important to understand their properties, generation mechanisms, and the effects they have in the plasma in order to extrapolate to inaccessible regions of space.

## Figures and Tables

**Table 1:** Measurements and Calculations for the Intervals of the Emission Observed on 1 December 2015 at 4:51:00.471 UT by MMS 3

MVA (Figure 3)	*θ*_*kB*_	*θ*_*kV*_	*ω*_*ce*_(Hz)	*n*_*e*_(cm^−3^)	k	*λ*_max_/*λ*_int_	*λ*_int_/*λ*_min_	*λ*_max_/*λ*_min_	B_0_ (nT)	V_*b*_ (km/s)
b	18.1°	77.2°	499.2	19.36	[0.354, −0.872, 0.339]	1.5	426.6	622.5	[−1.56, 17.38, −3.68]	[−52.1, −20.5, −45.8]
c	16.9°	80.1°	484.8	19.76	[−0.349, 0.889, −0.296]	1.1	2011.5	2129.2	[−1.93, 17.01, −2.61]	[−47.9, −21.2, −49.0]
d	27.1°	70.7°	489.8	19.75	[0.444, −0.794, 0.415]	1.0	1070.3	1099.8	[−2.60, 17.21, −1.84]	[−46.0, −22.1, −50.0]
e	1.3°	81.7°	488.3	19.83	[−0.185, 0.979, −0.090]	1.1	509.5	546.5	[−2.86, 17.13, −1.63]	[−44.5, −23.4, −49.4]
f	9.1°	88.7°	484.5	19.67	[−0.322, 0.940, −0.112]	1.1	1439.5	1570.8	[−3.00, 16.99, −1.36]	[−44.5, −22.8, −49.5]
g	55.8°	67.0°	488.6	19.75	[−0.058, −0.534, 0.844]	1.1	74.5	81.5	[−3.66, 17.03, −1.12]	[−45.1, −22.8, −50.6]
h	18.7°	81.9°	489.5	19.73	[0.515, −0.848, 0.129]	1.1	408.1	459.8	[−3.89, 17.01, −1.11]	[−46.0, −23.7, −52.0]
i	25.9°	85.8°	492.8	19.68	[−0.540, 0.817, 0.203]	1.4	387.7	540.0	[−3.92, 17.12, −1.23]	[−46.3, −24.0, −53.1]
j	5.4°	88.7°	484.6	19.67	[0.274, −0.949, 0.153]	1.0	1549.5	1595.1	[−3.41, 16.87, −1.85]	[−45.6, −23.5, −53.0]
k	17.9°	87.7°	486.2	19.66	[−0.006, 0.935, −0.354]	1.1	304.2	325.8	[−3.94, 16.75, −2.37]	[−45.1, −23.7, −53.3]
l	20.9°	88.1°	487.9	19.52	[0.029, 0.921, −0.388]	1.2	1585.9	1901.2	[−4.01, 16.79, −2.40]	[−45.1, −23.8, −53.4]
m	23.8°	88.2°	500.4	19.40	[−0.045, −0.909, 0.413]	1.4	1241.4	1775.5	[−4.61, 17.10, −2.44]	[−45.5, −24.7, −53.5]
n	27.7°	85.8°	508.0	19.20	[−0.008, −0.855, 0.518]	1.6	556.7	885.1	[−4.93, 17.27, −2.57]	[−46.2, −25.6, −53.6]
o	14.9°	85.2°	517.6	19.13	[−0.076, 0.953, −0.292]	1.1	202.4	225.8	[−5.32, 17.51, −2.66]	[−46.6, −26.8, −53.5]
p	20.2°	84.8°	520.3	19.04	[0.002, 0.937, −0.350]	1.9	61.7	116.0	[−5.37, 17.57, −2.81]	[−46.5, −27.1, −53.4]

*Note*. MMS = Magnetospheric Multiscale; MVA = minimum variance analysis.

## Key Points:

- In the observation frame the emissions have a wide range of frequencies, but in the rest frame they have < 70%that of the electron cyclotron
- Lion roars propagate at oblique angles in the magnetosheath, quasi-parallel on average, but in a very wide range of angles
- Lion roars are important in the regulation of magnetosheath electron anisotropies as they convect from the bow shock to the magnetopause

## References

[R1] Baumjohann W, Treumann RA, Georgescu E, Haerendel G, Fornacon K-H, & Auster U (1999). Waveform and packet structure of lion roars. Annales Geophysicae, 17(12), 1528–1534. 10.1007/s00585-999-1528-9

[R2] Breneman A, Cattell C, Schreiner S, Kersten K, Wilson LB, Kellogg P, (2010). Observations of large-amplitude, narrowband whistlers at stream interaction regions. Journal of Geophysical Research, 115, A08104 10.1029/2009JA014920

[R3] Breuillard H, Contel OL, Chust T, Berthomier M, Retino A, Turner DL, (2017). The properties of lion roars and electron dynamics in mirror-mode waves observed by the Magnetospheric MultiScale mission. Journal of Geophysical Research: Space Physics, 123, 93–103. 10.1002/2017JA024551

[R4] Brice N (1964). Fundamentals of very low frequency emission generation mechanisms. Journal of Geophysical Research, 69(21), 4515–4522. 10.1029/JZ069i021p04515

[R5] Chang O, Gary SP, & Wang J (2013). Whistler turbulence at variable electron beta: Three-dimensional particle-in-cell simulations. Journal of Geophysical Research: Space Physics, 118, 2824–2833. 10.1002/jgra.50365

[R6] Farris MH, Petrinec SM, & Russell CT (1991). The thickness of the magnetosheath: Constraints on the polytropic index. Geophysical Research Letters, 18(10), 1821–1824. 10.1029/91GL02090

[R7] Farris MH, & Russell CT (1994). Determining the standoff distance of the bow shock: Mach number dependence and use of models. Journal of Geophysical Research, 99(A9), 17,681–17,689. 10.1029/94JA01020

[R8] Gary SP, & Mellott MM (1985). Whistler damping at oblique propagation: Laminar shock precursors. Journal of Geophysical Research, 90(A1), 99–104. 10.1029/JA090iA01p00099

[R9] Hughes RS, Gary SP, &Wang J (2014). Electron and ion heating by whistler turbulence: Three-dimensional particle-in-cell simulations. Geophysical Research Letters, 41, 8681–8687. 10.1002/2014GL062070

[R10] Kennel C, & Petschek H (1966). Limit on stably trapped particle fluxes. Journal of Geophysical Research, 71, 1–28. 10.1063/1.1761629

[R11] Kivelson MG, & Southwood DJ (1996). Mirror instability II: The mechanism of nonlinear saturation. Journal of Geophysical Research, 101(A8), 17,365–17,371. 10.1029/96JA01407

[R12] Le Contel O, Leroy P, Roux A, Coillot C, Alison D, Bouabdellah A, (2016). The search-coil magnetometer for MMS. Space Science Reviews, 199(1–4), 257–282. 10.1007/s11214-014-0096-9

[R13] Lengyel-Frey D, Farrell WM, Stone RG, Balogh A, & Forsyth R (1994). An analysis of whistler waves at interplanetary shocks. Journal of Geophysical Research, 99(A7), 13,325–13,334. 10.1029/94JA00781

[R14] Lucek EA, Constantinescu D, Goldstein ML, Pickett J, Pinçon JL, Sahraoui F, (2005). The magnetosheath. Space Science Reviews, 118(1–4), 95–152. 10.1007/s11214-005-3825-2

[R15] Masood W, Schwartz SJ, Maksimovic M, & Fazakerley AN (2006). Electron velocity distribution and lion roars in the magnetosheath. Annales Geophysicae, 24(6), 1725–1735. 10.5194/angeo-24-1725-2006

[R16] Moullard O, Burgess D, & Bale SD (1998). Whistler waves observed during an in-situ solar type III radio burst. Astronomy and Astrophysics, 335,703–708.

[R17] Moullard O, Burgess D, Salem C, Mangeney A, Larson DE, & Bale SD (2001). Whistler waves, Langmuir waves and single loss cone electron distributions inside a magnetic cloud: Observations. Journal of Geophysical Research, 106(A5), 8301–8313. 10.1029/2000JA900144

[R18] Pollock C, Moore T, Jacques A, Burch J, Gliese U, Saito Y, (2016). Fast plasma investigation for magnetospheric multiscale. Space Science Reviews, 199(1–4), 331–406. 10.1007/s11214-016-0245-4

[R19] Russell CT, Anderson BJ, Baumjohann W, Bromund KR, Dearborn D, Fischer D, (2016). The magnetospheric multiscale magnetometers. Space Science Reviews, 199(1–4), 189–256. 10.1007/s11214-014-0057-3

[R20] Schwartz S (1998). Shock and discontinuity normals, mach numbers, and related parameters. ISSI Scientific Reports Series, 1, 249–270.

[R21] Silverman BW (1986). Density estimation for statistics and data analysis (Vol. 37, 120 pp.). London: Chapman and Hall 10.2307/2347507

[R22] Smith EJ, Holzer RE, & Russell CT (1969). Magnetic emissions in the magnetosheath at frequencies near 100 Hz. Journal of Geophysical Research, 74(11), 3027–3036. 10.1029/JA074i011p03027

[R23] Smith EJ, & Tsurutani BT (1976). Magnetosheath lion roars. Journal of Geophysical Research, 81(13), 2261–2266. 10.1029/JA081i013p02261

[R24] Torbert RB, Russell CT, Magnes W, Ergun RE, Lindqvist P-A, LeContel O, (2016). The FIELDS instrument suite on MMS: Scientific objectives, measurements, and data products. Space Science Reviews, 199(1–4), 105–135. 10.1007/s11214-014-0109-8

[R25] Tsurutani BT, Smith EJ, Anderson RR, Ogilvie KW, Scudder JD, Baker DN, & Bame SJ (1982). Lion roars and nonoscillatory drift mirror waves in the magnetosheath. Journal of Geophysical Research, 87(A8), 6060 10.1029/JA087iA08p06060

[R26] Vedenov AA, & Sagdeev RZ (1961). Some properties of plasma with an anisotropic ion velocity distribution in a magnetic field. Plasma Physics and the Problem of Controlled Thermonuclear Reactions, 3, 332–339.

[R27] Walker SN, Balikhin M. a., & Nozdrachev MN (1999). Ramp nonstationarity and the generation of whistler waves upstream of a strong quasiperpendicular shock. Geophysical Research Letters, 26(10), 1357–1360. 10.1029/1999GL900210

[R28] Wilson LB III, Koval A, Szabo A, Stevens ML, Kasper JC, Cattell CA, & Krasnoselskikh VV (2017). Revisiting the structure of low-Mach number, low-beta, quasi-perpendicular shocks. Journal of Geophysical Research: Space Physics, 122, 9115–9133. 10.1002/2017JA024352 30410850 PMC6219398

[R29] Wilson LB, Koval A, Szabo A, Breneman A, Cattell CA, Goetz K, (2013). Electromagnetic waves and electron anisotropies downstream of supercritical interplanetary shocks. Journal of Geophysical Research: Space Physics, 118, 5–16. 10.1029/2012JA018167

[R30] Zhang Y, Matsumoto H, & Kojima H (1998). Lion roars in the magnetosheath: The Geotail observations. Journal of Geophysical Research, 103(A3), 4615–4626. 10.1029/97JA02519

